# AuPt Bimetallic Nanozymes for Enhanced Glucose Catalytic Oxidase

**DOI:** 10.3389/fchem.2022.854516

**Published:** 2022-02-21

**Authors:** Feixiang Chen, Tianlin Song

**Affiliations:** ^1^ Shanghai Key Laboratory of Green Chemistry and Chemical Processes, School of Chemistry and Molecular Engineering, East China Normal University, Shanghai, China; ^2^ Tongji University Cancer Center, Shanghai Tenth People’s Hospital, Tongji University School of Medicine, Shanghai, China

**Keywords:** AuPt alloys, bimetallic nanozymes, electron transfer, catalytic activity, rational design

## Abstract

Au metal nanoparticles as artificial nanozymes have attracted wide interest in biotechnology due to high stability and easy synthesis. Unfortunately, its catalytic activity is limited by the uniform surface electron distribution, fundamentally affecting the oxidation efficiency of glucose. Here, we synthesized AuPt bimetallic nanoparticles with unique surface electron structure due to the coupling effect of the two metal components, achieving improved glucose catalytic oxidase. Because of the effective work function difference between the two metals in AuPt, the electrons will transfer from Au to accumulate on Pt, simultaneously contributing to the substantial enhancement of Au-induced glucose oxidase and Pt-induced catalase performance. We systematically studied the enzyme-catalytic efficiency of AuPt with varied two metal proportions, in which Au:Pt at 3:1 showed the highest catalytic efficiency of glucose oxidase in solution. The AuPt nanoparticles were further co-cultured with cells and also showed excellent biological activity for glucose oxidase. This work demonstrates that the physicochemical properties between different metals can be exploited for engineering high-performance metal nanoparticle-based nanozymes, which opens up a new way to rationally design and optimize artificial nanozymes to mimic natural enzymes.

## Introduction

Natural enzymes as biocatalysts, mediate almost every biological process in living, but their inherent disadvantages such as high cost, easy inactivation, and difficult to recover, largely limit application in biomedical engineering (Wolfenden and Snider, 2001; Pietrzak and Ivanova, 2021). This inspires scientists to explore artificial substitutes for enzymes (Wang et al., 2020). Many nanomaterials so far have been found with significant enzyme-like catalytic activities, such as Fe_3_O_4_ nanoparticles (NPs) and carbon nanotubes with peroxidase (POD-like) properties, and Pt NPs and CeO_2_ NPs with catalase (CAT-like) activities, which are commonly referred to as nanozymes (Gao et al., 2007; Huang et al., 2019). As an alternative to natural enzymes, nanozymes possess characteristics such as low cost, high stability, easy mass production, and adjustable activity, and show enormous potentials in a wide range of applications, including biosensing, imaging, food manufacturing, and pollution prevention (Dong et al., 2012; Fan et al., 2012; Tian et al., 2020). However, low catalytic efficiency is still one key issue facing in practical applications (Chen et al., 2021).

Gold nanoparticles (Au NPs) with glucose oxidase (GOx-like) features have yielded promising responses in biomedical application (Lin et al., 2014; Wu et al., 2018; Zhang et al., 2019a). In the relevant studies of structure-performance of Au nanozymes, a variety of parameters including size and shape, surface structure, local composition and chemical bonding have been proved to play important impacts on their catalytic performances (Zhou et al., 2010; Hakkinen, 2012; Shen et al., 2015; Li et al., 2019; Lou-Franco et al., 2020). In fact, during catalytic process, the oxidation-redox reactions take place just on the surface of Au NPs, realized by the electron transfer between Au and the surrounding reactants (Della Pina et al., 2008). Therefore, regulating the physicochemical properties of Au NPs is vitally required for effective modulation of electron transfer on surface, so as to boost their intrinsic biomimetic catalytic ability (Jiao et al., 2019). On account of the effective work function difference between different composites, it offers the possibility to modulate the electron density of Au at the atomic scale, which may favor the fast mass transport and electron transfer during the catalytic process (Yamauchi et al., 2012). Therefore, the design of Au with other composites is greatly expected to be an effective way to develop Au nanozymes with excellent performance, and even may bring unprecedented insights into the relationship between electron density of Au and enzyme-like activities.

Herein, AuPt bimetallic alloys were synthesized as effective nanozymes for the catalytic oxidation of glucose. Since the work function of Au was less than that of Pt in AuPt alloys, the electrons on Au will flow to accumulate on Pt, making Pt electron-rich. This rearrangement of electrons in AuPt contributes to enhanced catalytic oxidation performance of Au for GOx, as well as catalytic reduction of H_2_O_2_ by Pt, thus giving 1.4-fold GOx-like activity improvement over Au NPs alone. AuPt alloys showed negligible cytotoxicity at a certain concentration, but significantly affected the energy metabolism process of cells due to the consumption of glucose. It was expected to use AuPt bimetallic nanozymes for subsequent biological researches, such as treatment of cancer or diabetes (Nosrati et al., 2021).

## Materials and Methods

### Materials

HAuCl_4_.3H_2_O (≥49.0% Au basis), H_2_PtCl_6_.6H_2_O (≥37.50% Pt basis), hydrogen peroxide (H_2_O_2_, 30%) and Methyl thiazolyl tetrazolium (MTT, 98%) were purchased from Sigma-Aldrich (Louis, MO, United States). Glucose was purchased from Macklin. Trifluoroacetic acid (CF_3_COOH, 99%) was purchased from Alfa Aesar. The ATP assay kit, Enhanced BCA protein assay kit and Calcein/PI cell viability assay kit were from Beyotime Institute of Biotechnology, China. All chemical agents in this work were utilized without further purification. The Milli-Q water was obtained from the Milli-Q System.

### Characterization of AuPt Alloys

The transmission electron microscope (TEM) images of the nanoparticles were obtained on an FEI Tecnai G2 F30 microscope. The crystalline phases of the materials were collected with X-ray powder diffraction (XRD; Rigaku Ultima Ⅳ), using copper Kɑ radiation (*λ* = 0.154056 nm) in the 2θ range of 20°–90° at a scan rate of 20°/min. Fourier transform infrared spectroscopy (FT-IR) spectra was measured by a BRUKER TENSOR Ⅱ in the range of 4,000–400 cm^−1^. Dynamic light scattering (DLS) particle size analyzer (Malvern, United States) was used to measure the hydrophilic diameters of the particles. The concentration of Au and Pt was measured by Agilent Technologies 5100 inductively coupled plasma optical emission Spectrometry (ICP-OES). Shimadzu, AXIS SUPRA was used to detect X-ray photoelectron spectroscopy (XPS). The position of the C 1s peak at 284.8 eV was used as a calibration reference to determine the accurate binding energies. UV-vis absorption spectra was recorded on Shimadzu UV 3600 plus. The oxygen content in water was measured by dissolved oxygen tester JPSJ-605F, Leici China. The confocal laser scanning microscopy (CLSM) images were obtained using NIKON A1 R.

### Synthesis of AuPt Alloys

We added 5 ml of HAuCl_4_.3H_2_O (20 mmol) and H_2_PtCl_6_.6H_2_O (20 mmol) in different volume ratios to 95 ml water, stirred it for 5 min until it was evenly mixed, and then heated the mixture to boiling. Then, 10 ml sodium citrate aqueous solution with a concentration of 20 mg/ml was added to the boiling liquid and stirred for 10 min. Next, turned off the heat and continued to stir vigorously to room temperature. AuPt alloys with different proportions were obtained by centrifugal washing. Au NPs and Pt NPs were synthesized by similar methods. The composition of AuPt alloys was determined by ICP-OES.

### Glucose Oxidation Reaction

The catalytic performance of the alloys was characterized by the formation of gluconic acid. 50 μL AuPt alloys (2 mg/ml) and 950 μL glucose (1 M) were mixed evenly for 4 h to verify the effect of AuPt alloys on glucose catalytic oxidation. After the reaction, AuPt alloys were removed by centrifugation to obtain supernatant containing gluconic acid.

Solution A (5 mM EDTA and 0.15 mM triethylamine aqueous solution), solution B (3 M hydroxylamine, NH_2_OH), and solution C (1 M HCl, 0.1 M FeCl_3_ and 0.25 M CCl_3_COOH aqueous solution) were respectively prepared. Then, 250 μL solution A and 25 μL solution B were successively added to the clear liquid. After 25 min of incubation, 125 μL solution C was added, and the mixture was evenly mixed for 5 min before spectral test (Huang et al., 2017).

### Catalyze Decomposition of H_2_O_2_

10 μL AuPt alloys (2 mg/ml) were mixed with an aqueous solution of H_2_O_2_ (10 mM), with a total volume of 5 ml, and the amount of oxygen produced by the decomposition of H_2_O_2_ was recorded by a dissolved oxygen recorder (Luo et al., 2021). All the reactions were carried out in deoxidized water at 37 °C.

### Cell Viability Assessment for AuPt Alloys

In order to evaluate the cell compatibility of AuPt alloys, PC12 cells were seeded into 96-well cell culture plates at 1×10^4^/well for 24 h. Then Au_0.75_Pt_0.25_ alloys with different concentrations were added into 96-well plates and cultured for 24 h. After the culture medium was sucked out, 100 μL MTT (phosphate buffered saline (PBS) buffer solution 0.6 mg/ml) was added to each well and cultured for 4 h. Finally, removed the MTT solution, added 100 μL dimethyl sulfoxide (DMSO), and incubated for 10 min. The absorbance of the solution at 490 nm was measured with a microplate reader.

### Confocal Laser Scanning Microscopy Imaging

PC12 cells were inoculated in 1 ml CLSM dish at 1×10^5^/ml and incubated overnight to ensure firm cell adhesion. The cells were incubated with AuPt alloys of different concentrations for 24 h, and then the cells in the corresponding groups were dyed alive and dead with Calcein (green) and PI (red).

### Intracellular ATP Detection

PC12 cells were inoculated at a density of 5 × 10^5^ cells/well and incubated in 6-well plates for 12 h. Then AuPt alloys with different proportions were added and incubated for 24 h. After that, the cells were lysed with ATP lysis buffer, and the supernatant was collected by centrifugation. The protein concentration of each sample was determined by BCA protein concentration kit, and ATP concentration was detected by ATP detection kit (Liu et al., 2021).

## Results and Discussions

### Characterization of Material Properties

**FIGURE 1:**
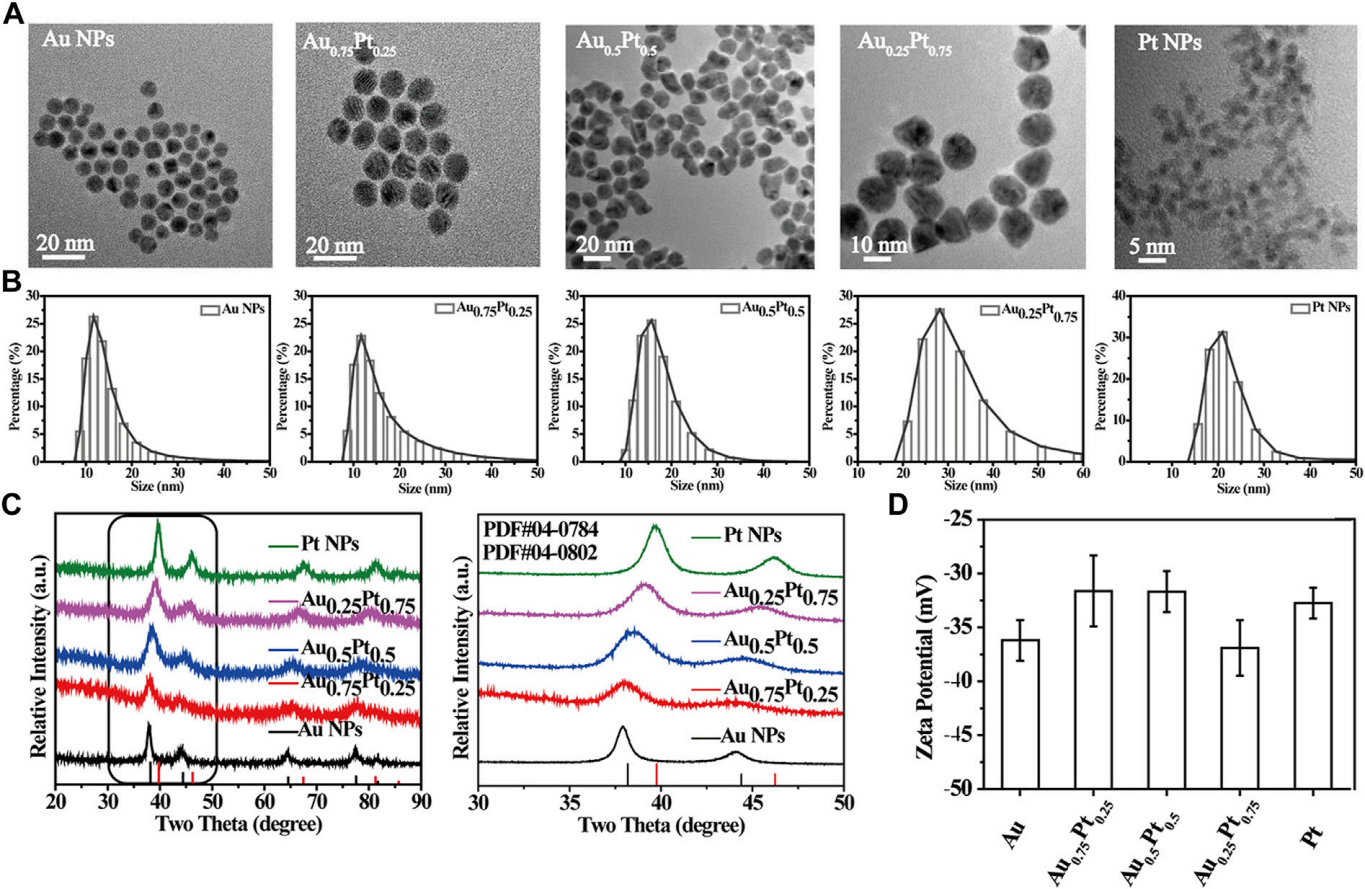
Characterization of the prepared AuPt alloys. **(A)** TEM images of AuPt alloys with different proportions. **(B)** Hydrodynamic diameter distribution of AuPt alloys with different proportions. **(C)** XRD patterns of AuPt alloys with different proportions, partial enlarged XRD patterns. **(D)** Zeta potential of AuPt alloys with different proportions.

AuPt alloys with different proportions were prepared by one-step reduction of AuCl_4_^−^ and PtCl_6_^2−^ with sodium citrate in water. As shown in Figure 1A, TEM images showed that these AuPt alloys with different proportions were uniformly spherical. High resolution TEM (HRTEM) images and the corresponding FAST Fourier transform (FFT) further revealed the lattice plane of the Au_0.75_Pt_0.25_ alloys (Supplementary Figure S1). The calculated lattice spacing of the (111) plane of the alloys was 0.230 nm, which was between the lattice spacing of the pure Au (111) plane (0.235 nm) and that of the pure Pt (111) plane (0.228 nm). The lattice spacing in the middle proved the formation of AuPt alloys (He et al., 2017).

In addition, the average hydrodynamic diameters of AuPt alloys synthesized with different AuPt ratios by DLS test were approximately 10–20 nm and had good dispersibility (Figure 1B) (Zhang et al., 2019b). The stability of the Au_0.75_Pt_0.25_ was further evaluated. TEM images showed that the morphological characteristics of Au_0.75_Pt_0.25_ did not change significantly after 3 days of soaking in PBS (Supplementary Figure S2), indicating that Au_0.75_Pt_0.25_ had excellent stability. The Zeta potential was about -30 mV due to the -COOH in sodium citrate on the surface of the AuPt alloys (Figure 1D). Supplementary Figure S3 showed the characteristic peaks of sodium citrate, such as –C=O characteristic absorption peak at 1,600 cm^−1^ and -CH_2_ shear vibration peak at 1,400 cm^−1^, indicating the successful preparation of sodium citrate stabilized AuPt alloys (Sanches et al., 2011; Zhang et al., 2018).

It was well known that the lattice of two metals would change after they formed alloys, and their diffraction patterns would change correspondingly according to the basic principle of crystal diffraction. Therefore, XRD pattern was used to further confirm the formation of AuPt alloys. Figure 1C showed XRD patterns of alloys with different proportions. Diffraction peaks of Au, Pt and AuPt alloys with different AuPt ratios indicated that the prepared samples all had face-centered cubic (fcc) phases. According to Figure 1C, the 2θ peaks (111) and (200) of AuPt NPs fell between the corresponding 2θ peaks of Au and Pt NPs (Liu et al., 2011). With the change of AuPt ratios, the positions of these peaks changed gradually, indicating the change of alloying degree. UV-vis absorption spectrum (Supplementary Figure S4) also confirmed the formation of AuPt alloys. With the gradual addition of Pt component, the typical absorption of Au NPs at 520 nm due to surface plasmon resonance would gradually disappear. The vanishing peak also represented the gradual formation of AuPt alloys (Zhao et al., 2014).

The relative abundance of Au and Pt in AuPt alloys detected by ICP and XPS was shown in Supplementary Table S1. Because the standard redox potential E^0^ for AuCl_4_^−^/Au^0^ couple (+0.99 V) was higher than that of PtCl_6_^2−^/Pt^0^ couple (+0.74 V), the Au was reduced first (De and Rao, 2005). The percentage of Au element in the alloys obtained from ICP was slightly higher than that in the raw solution, and the results of XPS measurement further confirmed that Pt element was enriched on the surface of the alloys.

### AuPt Alloys for Glucose Catalytic Oxidation and Decomposition of H_2_O_2_

**FIGURE 2:**
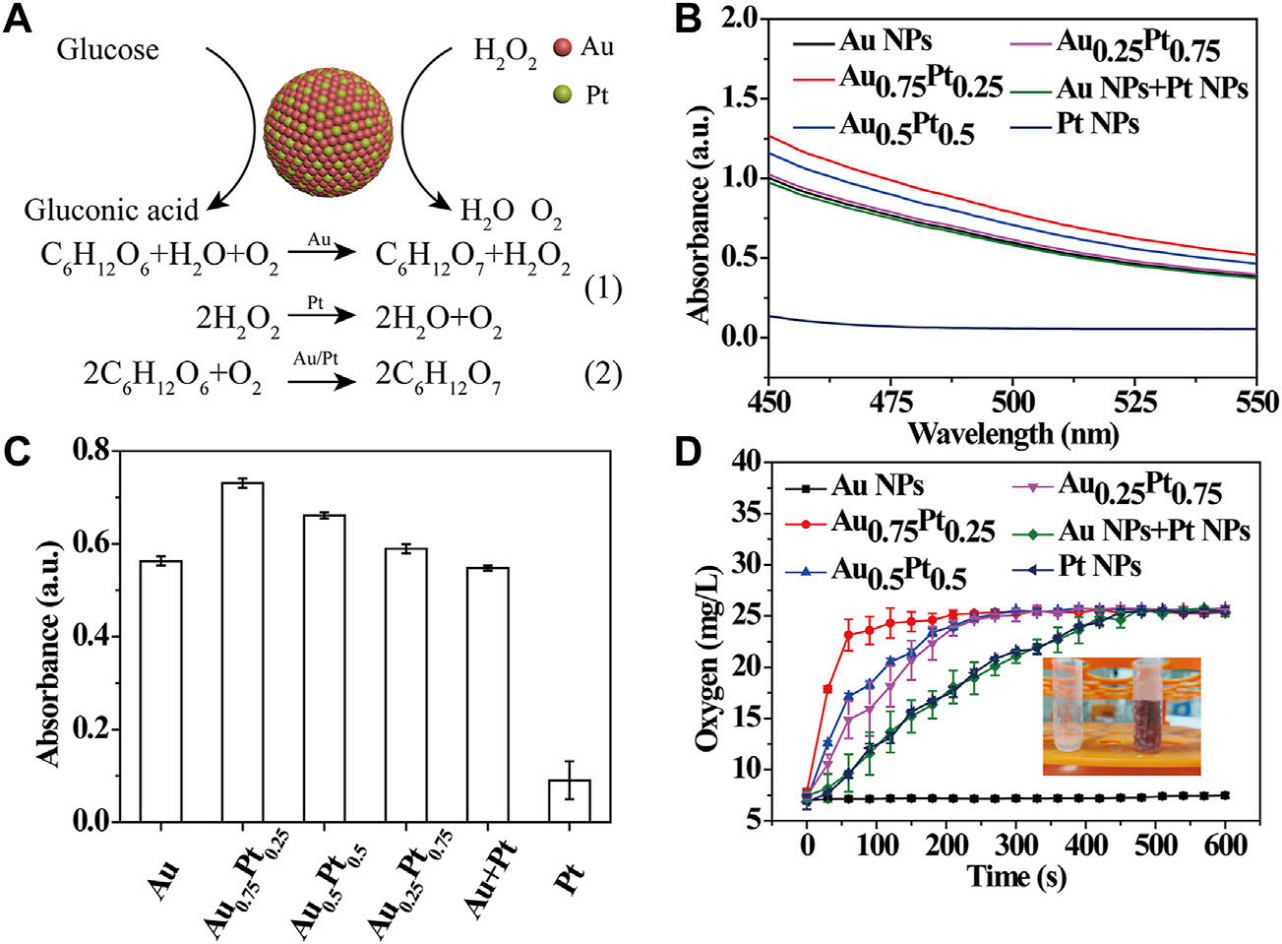
Catalytic activity test of AuPt alloys. **(A)** Schematic illustration of catalytic oxidation of glucose in AuPt Alloys. **(B)** UV-vis absorption spectra of AuPt alloys with different proportions incubation with glucose for 4 h. AuPt alloys catalyzed the oxidation of glucose to gluconic acid, which is mixed with hydroxylamine and Fe^3+^ to produce a red complex. **(C)** Absorbance value at 505 nm determined by a colorimetric assay. **(D)** Dissolved oxygen level of AuPt alloys with different proportions solution after incubation with H_2_O_2_.

Since the prepared AuPt alloys included different catalytic properties, in which Au components possessed the intrinsic GOx-like activity (Luo et al., 2010), while Pt components owned the intrinsic CAT-like activity [Figure 2A(1)] (Fan et al., 2011), it was expected that AuPt alloys could catalyze the cascade reaction of glucose oxidation [Figure 2A(2)]. To prove it, the GOx-like activity of AuPt alloys was first investigated. AuPt alloys in different proportions reacted with O_2_ in the reaction solution to catalyze the oxidation of glucose, resulting in H_2_O_2_ and gluconic acid. The reaction solution was centrifuged to remove AuPt alloys, and the supernatant obtained contained H_2_O_2_ and gluconic acid. In a typical experiment, with gluconic acid as substrate, NH_2_OH and Fe^3+^ were successively added to the solution, and the reaction of glucose to gluconic acid catalyzed by AuPt alloys were detected by a colorimetric assay. As shown in Figure 2B, the apparent absorbance band at 450–550 nm confirmed that gluconic acid was the product of glucose oxidation catalyzed by AuPt alloys. Next, we made statistics on the absorbance value at 505 nm (Figure 2C), and it could be seen that Au_0.75_Pt_0.25_ had the best catalytic effect compared with other alloys and physical mixing group. The above experimental results confirmed the GOx-like activity of the AuPt alloys.

Then, the CAT-like activity of AuPt alloys was studied. Since it could catalyze H_2_O_2_ to produce H_2_O and O_2_, we explored the catalytic process of AuPt alloys for H_2_O_2_ by testing the content of dissolved oxygen. A large number of bubbles were observed in the tubes containing H_2_O_2_ after the addition of AuPt alloys in different proportions (except pure Au NRs), demonstrating that Pt in AuPt alloys could play a CAT-like effect (Figure 2D). Next, we used a solution-oxygen meter to further monitor the oxygen generation process and found that Au_0.75_Pt_0.25_ catalyzed the decomposition of H_2_O_2_ to generate oxygen at the fastest rate.

As mentioned above, AuPt alloys had GOx-like and CAT-like activities, and different AuPt ratios exhibited different nanozymes catalytic activities, among which Au_0.75_Pt_0.25_ had the best catalytic effect.

### Electron States in AuPt Alloys

**FIGURE 3:**
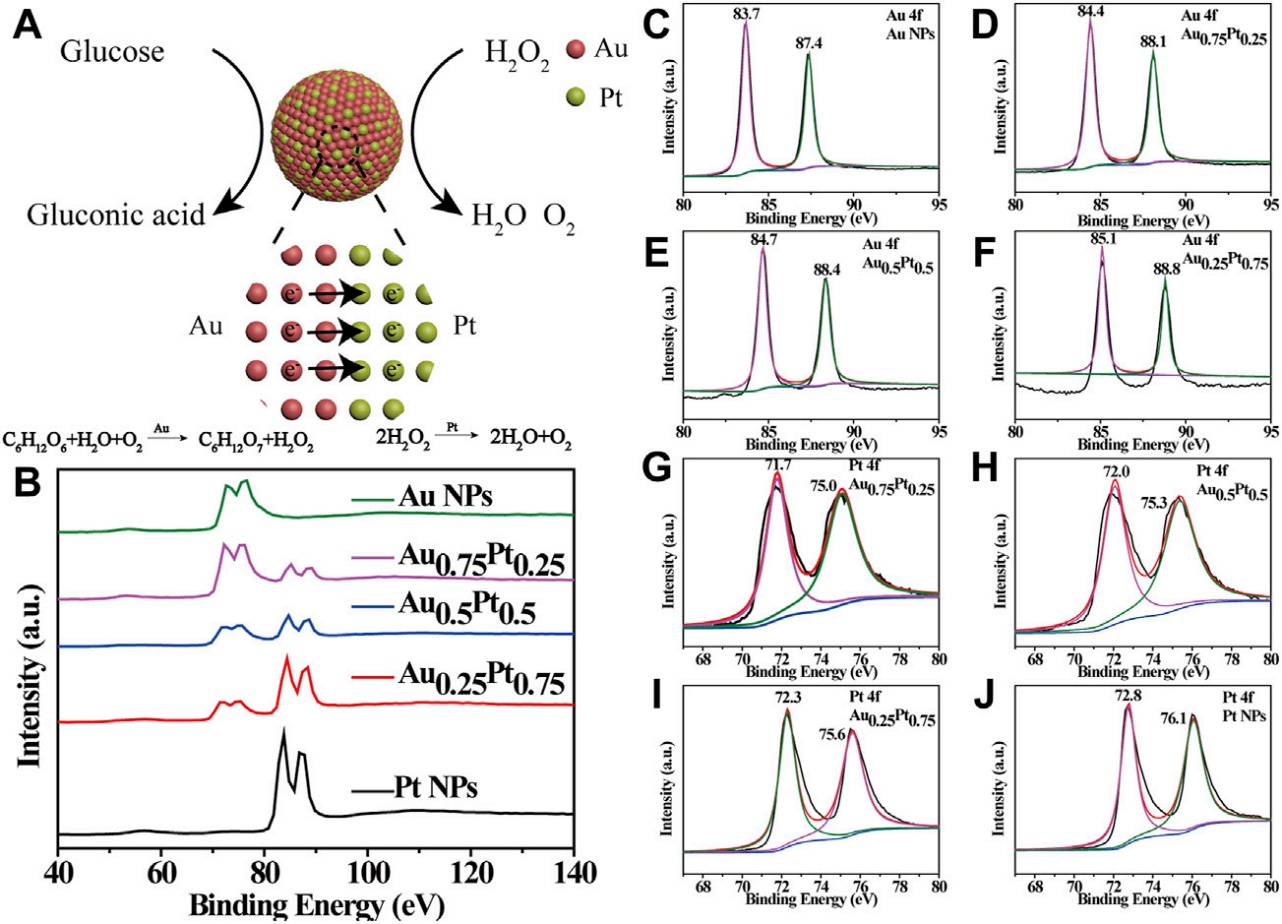
Electron distribution states in AuPt alloys. **(A)** Schematic illustration of electron transfer between Au and Pt. **(B)** Partial XPS spectrum of AuPt alloys with different proportions. Show the representative Au 4f of the **(C)** Au NPs, **(D)** Au_0.75_Pt_0.25_, **(E)** Au_0.5_Pt_0.5_, and **(F)** Au_0.25_Pt_0.75_. Show the representative Pt 4f of the **(G)** Au_0.75_Pt_0.25_, **(H)** Au_0.5_Pt_0.5_, **(I)** Au_0.25_Pt_0.75_ and **(J)** Pt NPs.

To further explore the reasons for the enhancement of the catalytic activity of AuPt alloys as nanozymes, we conducted the following analysis. We realized that it has been reported that the electronic structure of metal nanoparticles played a critical role in their catalytic activity (Duchesne et al., 2018). Therefore, we investigated whether the alloying of Au and Pt would change the electronic structures of Au and Pt components, thus affecting the catalytic performance (Figure 3A). The electron binding energies of XPS for Au and Pt in AuPt alloys with different proportions were used to reflect the charge distribution of AuPt in alloys (Supplementary Figure S5). Among them, in AuPt alloys system, the peak between 60 and 90 eV corresponded to Pt 4f_7/2_, Pt 4f_5/2_ and Au 4f_7/2_, Au 4f_5/2_, respectively (Figure 3B) (Yamauchi et al., 2012). As shown in Figure 3C, the binding energy of Au 4f in Au NPs was 83.7 and 87.4 eV, respectively. With the addition of Pt component, the 4f binding energy of Au component increased in AuPt alloys (Figures 3D–F). However, the 4f binding energy of Pt component in Pt NPs was 72.8 and 76.1 eV (Figure 3J), and the binding energy shifted to lower with the decrease of Pt component content (Figures 3G–I). The above analysis results proved that with the formation of AuPt alloys, the electron on Au component in AuPt alloys transfered to Pt component, resulting in lower electron density of Au component in AuPt alloys than Au NPs, and higher electron density of Pt component than Pt NPs.

Based on the above interesting results, we provided a theoretical basis for the change of electronic structure of AuPt alloys with different components. For bimetallic alloys, the work function of different metals was the decisive factor affecting the regulation of electronic structure. Then, for AuPt alloys, the work functions of Au and Pt were 5.54 and 6.13 eV, respectively. The difference in the work function between Au and Pt components caused the electrons on Au surface to be transfer towards Pt. Coincidentally, in the current study on the mechanism of Au nanozymes catalyzing glucose oxidation, Au components needed to maintain positive valence, and Pt surface was rich in electrons, which was conducive to its catalase properties. Therefore, electron transfer due to work function would synergistically enhance the effect of the two components of AuPt as nanozymes.

### *In vitro* Performance of AuPt Alloys

**FIGURE 4:**
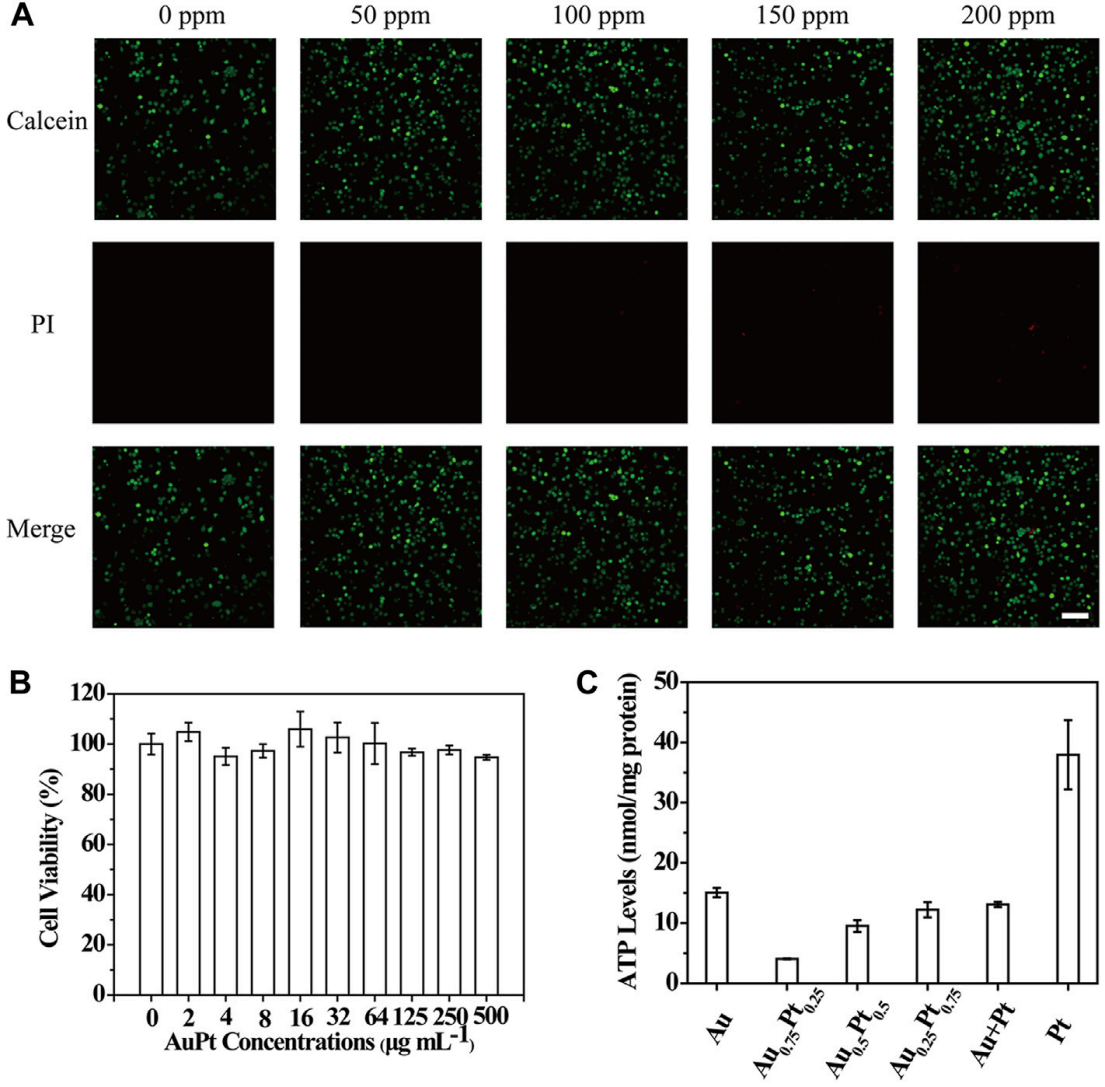
*In vitro* performance of AuPt alloys. **(A)** PC12 cells treated with different concentrations of Au_0.75_Pt_0.25_. **(B)** PC12 cells with Au_0.75_Pt_0.25_ treatment stained with Calcein (green, live cells) and PI (red, dead cells) for live/dead cell assay. **(C)** The intracellular ATP content of PC12 cells incubated with various AuPt alloys. The scale bar is 10 μm.

In order to be applied in subsequent biological studies, we evaluated the biological activity of AuPt alloys. MTT assay (Figure 4B) was used to verify the toxicity of PC12 cells co-cultured with AuPt alloys, and it was found that AuPt alloys had no obvious toxicity for cells. Subsequently, the survival of cells co-cultured with AuPt alloys for 24 h was observed in confocal images by live/dead staining experiment. Cells were stained with calcein/PI, in which living cells were stained green with calcein and dead cells were stained red with PI. Confocal images (Figure 4A) could be observed that AuPt alloys materials with different concentrations had no obvious killing effect on cells, which also facilitated the application of AuPt alloys for further research.

Encouraged by the high catalytic performance of AuPt alloys in aqueous solution for glucose and H_2_O_2_, we carried out cell level experiments to observe the catalytic effect of AuPt alloys. We verified whether AuPt alloys could affect the cellular metabolism process because glucose was the main energy source for cell activity. Intracellular ATP levels were measured using ATP detection kits (Supplementary Figures S6, S7). As shown in Figure 4C, by catalyzing glucose oxidation, AuPt alloys could effectively reduce intracellular ATP levels by affecting energy supply. And it was worth noting that Au_0.75_Pt_0.25_ also had the best catalytic effect at the cellular level. Therefore, AuPt alloys, which affected cell energy metabolism, were considered as a treatment for glucose-related diseases.

## Conclusion

In summary, our work reported the synthesis of AuPt alloys with varied molar ratios of AuPt via one-step reduction method in aqueous solution. This method provides a useful reference for the preparation of other binary or multicomponent metal nanoparticles. Since the large difference of the work function between Au and Pt, the electron transfer effect would occur in AuPt alloys, promoting the electron flow from the lower work function of Au to the higher work function side of Pt. Compared with pure Au and Pt, the alloying nanozymes showed higher activities of GOx-like and CAT-like. In addition, AuPt alloys showed low cytotoxicity *in vitro* but could affect the glucose metabolism due to its catalytic oxidation of glucose, holding great promise in the further development for clinic disease diagnosis and treatment.

## Data Availability

The original contributions presented in the study are included in the article/Supplementary Material, further inquiries can be directed to the corresponding author.
