# Supplementary material for: AuPt Bimetallic Nanozymes for Enhanced Glucose Catalytic Oxidase
Source: Front Chem. 2022 Feb 21;10:854516. doi: 10.3389/fchem.2022.854516 (PMC8899206; doi:10.3389/fchem.2022.854516)
Supplement: Supplementary file 1 [file DataSheet1.docx]

Supplementary Material

## Supplementary Figures

##
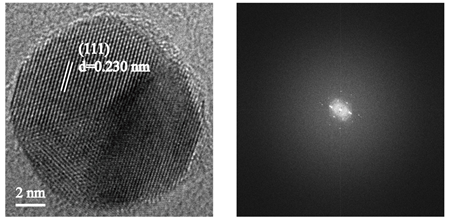


**Supplementary Figure 1.** HRTEM images and corresponding FFT images of Au_0.75_Pt_0.25_.

**
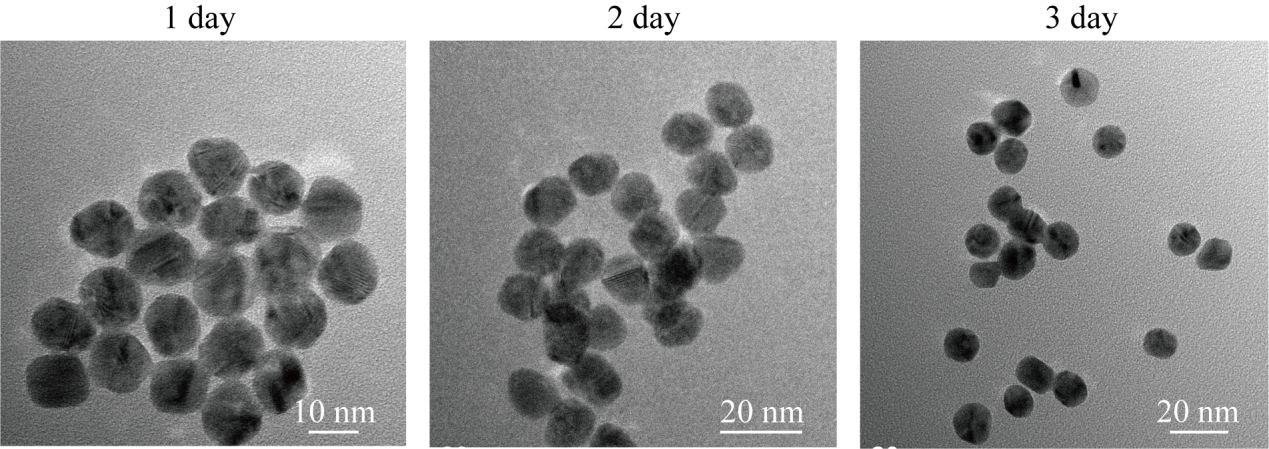
**

**Supplementary Figure 2.** The aqueous stability of Au_0.75_Pt_0.25_. TEM of Au_0.75_Pt_0.25_ in 1 day, 2 day and 3 day.


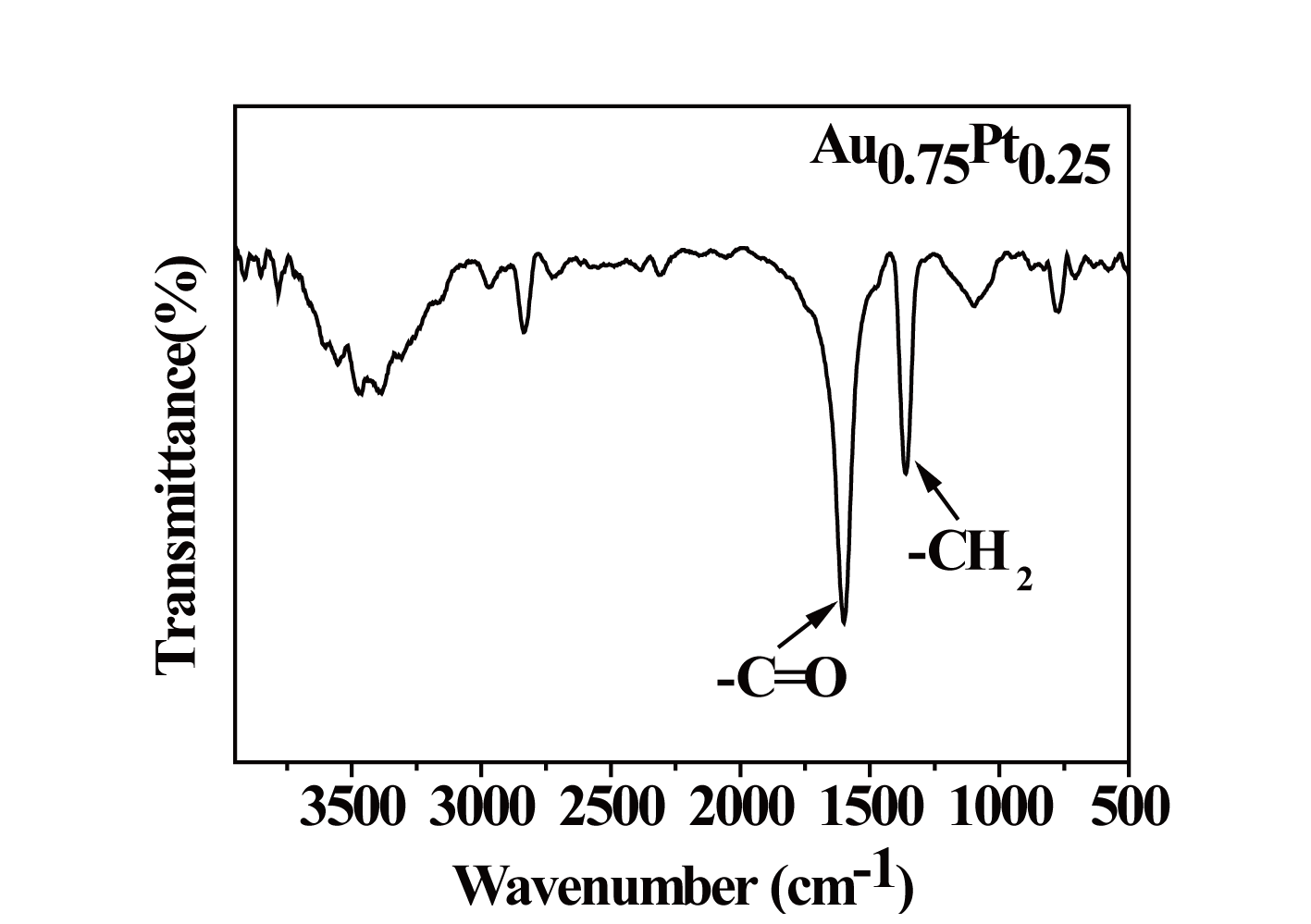


## Supplementary Figure 3. FT-IR spectra of Au_0.75_Pt_0.25_.


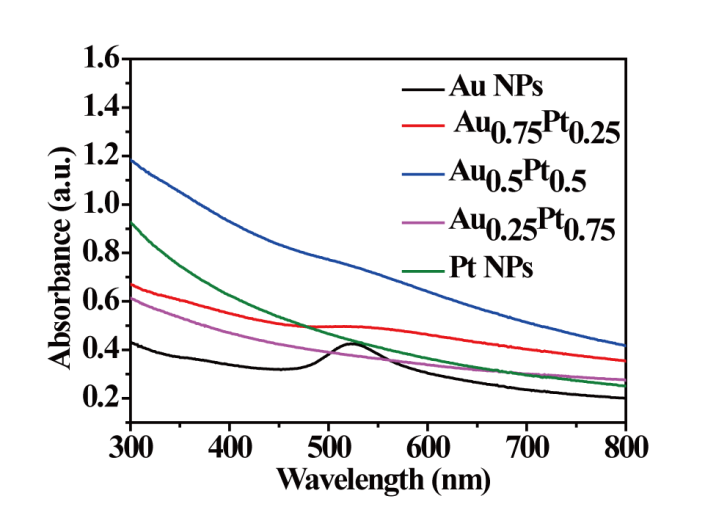


**Supplementary Figure 4.** UV-Vis spectrum of AuPt alloys with different proportions. The characteristic absorption of Au disappeared with the increase of Pt contents in AuPt alloys.


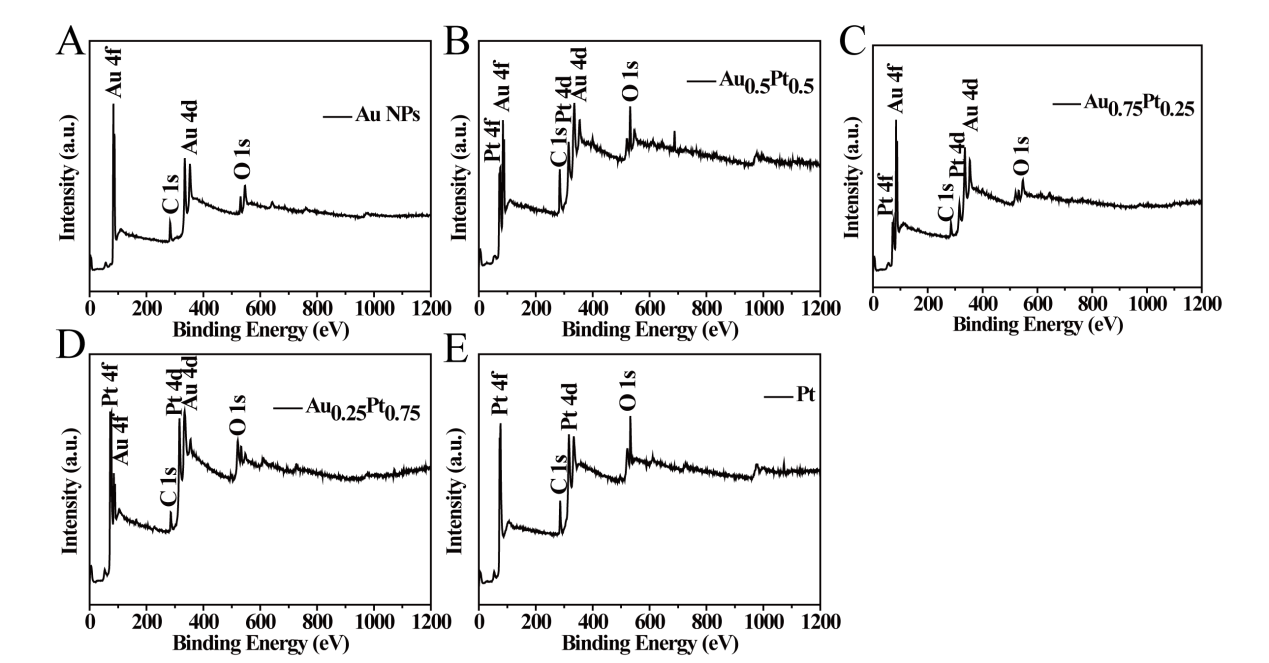


**Supplementary Figure 5.** XPS spectrum of AuPt alloys with different proportions.


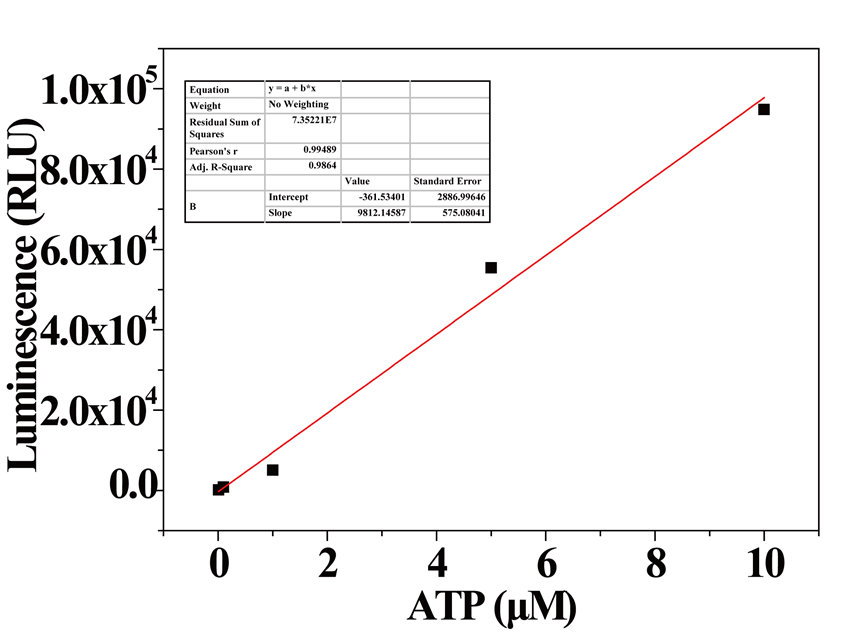


**Supplementary Figure 6.** ATP content standard curve.


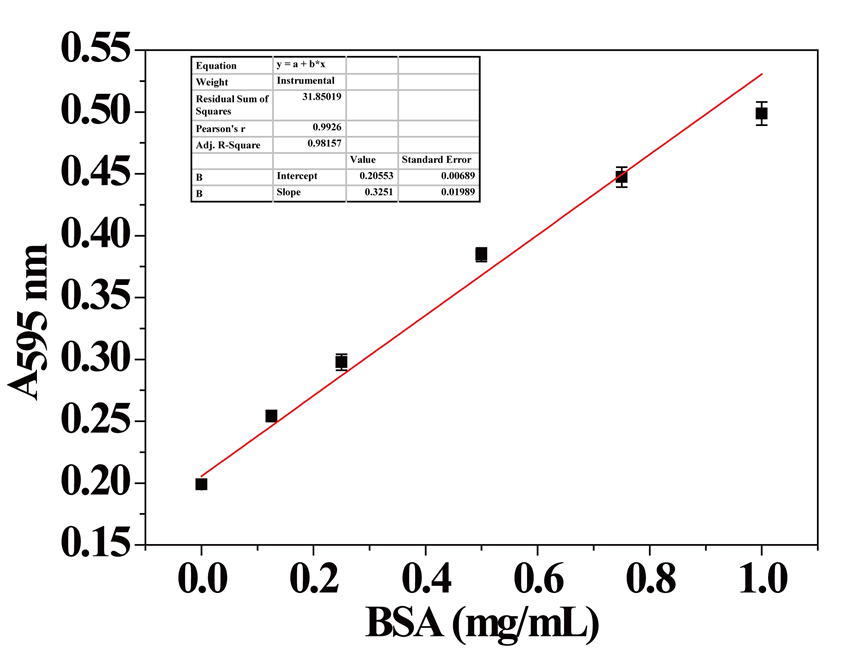


**Supplementary Figure 7.** Standard curve of BSA protein content.

| Feeding  Solutions  (Au:Pt) | 100:0 | 75:25 | 50:50 | 25:75 | 0:100 |
| --- | --- | --- | --- | --- | --- |
| ICP  (Au:Pt) | 100:0 | 79:21 | 57:43 | 35:65 | 0:100 |
| XPS  (Au:Pt) | 100:0 | 72:28 | 47:53 | 18:82 | 0:100 |

**Supplementary** **Table 1**. The relative abundance of Au and Pt in AuPt alloys detected by ICP and XPS.
